# Variation in symbiont density is linked to changes in constitutive immunity in the facultatively symbiotic coral, *Astrangia poculata*

**DOI:** 10.1098/rsbl.2022.0273

**Published:** 2022-11-16

**Authors:** Isabella Changsut, Haley R. Womack, Alicia Shickle, Koty H. Sharp, Lauren E. Fuess

**Affiliations:** ^1^ Texas State University, San Marcos, TX, USA; ^2^ Roger Williams University, Bristol, RI, USA

**Keywords:** symbiosis, ecoimmunology, invertebrate immunity, coral biology

## Abstract

Scleractinian corals are essential ecosystem engineers, forming the basis of coral reef ecosystems. However, these organisms are in decline globally, in part due to rising disease prevalence. Most corals are dependent on symbiotic interactions with single-celled algae from the family Symbiodiniaceae to meet their nutritional needs, however, suppression of host immunity may be essential to this relationship. To explore immunological consequences of algal symbioses in scleractinian corals, we investigated constitutive immune activity in the facultatively symbiotic coral, *Astrangia poculata*. We compared immune metrics (melanin synthesis, antioxidant production and antibacterial activity) between coral colonies of varying symbiont density. Symbiont density was positively correlated to both antioxidant activity and melanin concentration, likely as a result of the dual roles of these pathways in immunity and symbiosis regulation. Our results confirm the complex nature of relationships between algal symbiosis and host immunity and highlight the need for nuanced approaches when considering these relationships.

## 1. Introduction

Scleractinian corals are key ecosystem engineers, which create the structural basis of diverse coral reef systems [1]. However, the health of coral reefs worldwide is deteriorating, largely due to anthropogenic climate change [2]. Changing environmental conditions such as increased ocean temperatures and ocean acidification have led to coral die-offs [3]; global coral reef cover has declined by 50% from 1957 to 2007 [4]. The two largest drivers of coral mortality have been disease outbreaks and bleaching events [5–7]. Previous studies suggest extensive inter- and intraspecific variation in response to disease [8] and propensity to bleaching [9]. However, while the factors contributing to variation in bleaching susceptibility have been well studied in many coral species [9,10], the mechanisms driving variation in coral disease susceptibility largely remain unknown.

The coral immune response consists of pathogen recognition, signalling pathways, and effector responses [11]. Corals have a variety of pathogen recognition molecules, such as Toll-like receptors and NOD-like receptors, capable of identifying a diversity of pathogens [12]. Post-recognition, signalling pathways appropriate defence mechanisms and trigger effector responses [12]. Corals use effector responses such as melanin production, antioxidants and/or antimicrobial peptides to eliminate pathogens [12]. Preliminary evidence suggests that natural variation in several immune components might contribute to variation in disease resistance [13–15].

Beyond its role in pathogenic defence, the coral immune system also plays roles in the establishment and maintenance of symbioses [16–25]. The onset and maintenance of coral symbiosis with Symbiodiniaceae is theorized to circumvent or modulate the host immune response [19,25–28]. Furthermore, modification of immunity may extend beyond establishment of the relationship. In the threatened Caribbean coral *Orbicella faveolata*, which is obligately symbiotic, experimentally manipulated higher Symbiodiniaceae density was linked to negative effects on host immune gene expression [29]. Similarly, a study of *Acropora cervicornis*, found a negative correlation between bleaching and disease, suggesting the reduction in symbiont density associated with bleaching might reduce symbiont-associated immune suppression and increase host capacity to respond to disease [27]. Still understanding of the prevalence of potential symbiosis–immune trade-offs across cnidarian species, and the effects of natural symbiont density variation (i.e. non-stress related) on these trade-offs, is poorly understood. To better understand how Symbiodiniaceae density and immunity might be linked in diverse scleractinian corals, we investigated variation in constitutive immunity among colonies of the facultatively symbiotic scleractinian coral, *Astrangia poculata,* which displays immense natural variability in densities of its symbiont *Breviolum psygmophilum*.

## 2. Material and methods

### (a) Sample collection

*Astrangia poculata* colonies were collected from Fort Wetherill in Jamestown, Rhode Island in April 2021 (41°28′40″ N, 71°21′34″ W) at a depth of 10–15 m, via SCUBA. Colonies were visually assessed and sorted into either high or low symbiont density groups (termed ‘brown’ or ‘white’ colonies respectively); 10 colonies of each type were collected. Visual assessment of colony colour is a reliable method for distinguishing corals with high symbiont density (greater than 10^6^ cells cm^−2^) from those with low symbiont density (10^4^–10^6^ cells cm^−2^ [30]). It should be noted that we use the terms ‘brown’ and ‘white’ as colonies grouped in the white category are rarely completely aposymbiotic. Following collection, the colonies were returned to Roger Williams University (Bristol, RI) where they were maintained for several weeks in closed, recirculating systems containing locally sourced seawater and fed three times weekly with frozen copepod feed. This period allowed corals to acclimatize to common garden conditions, reducing the effect of environmental variation on our measured variables. Samples were then flash frozen in liquid nitrogen and shipped to Texas State University for analyses.

### (b) Protein extraction

Tissue was removed from colonies with extraction buffer (TRIS with DTT, pH 7.8) using protocols outlined by Fuess [31]. First, tissue was removed and isolated from a fixed surface area (2.14 cm^2^) on the flattest portion of the coral for Symbiodiniaceae density calculation. Then, tissue from the remaining fragment was removed and isolated into a separate aliquot. Both aliquots of tissue extracts were homogenized using a Fisherbrand Homogenizer 150 prior to downstream processing.

The Symbiodiniaceae aliquot was processed using a series of consecutive centrifugation and wash steps. The homogenate was centrifuged at 376 RCF for 3 min and the supernatant was removed. The resultant pellet was resuspended in 500 µl of deionized water, and the product was centrifuged again using the same procedure. This step was repeated, and the sample was preserved in 500 µl of 0.01% SDS in deionized water, stored at 4°C.

The host aliquot was processed to obtain subsamples for protein activity assays and melanin concentration estimation. Following homogenization, 1 ml of the host aliquot was flash frozen, and stored at 20°C for melanin concentration estimation. The remainder of the host aliquot was centrifuged for 5 min at 1301 RCF using an Eppendorf Centrifuge 5804 R. The resulting supernatant (protein-enriched cell-free extract) was flash frozen in liquid nitrogen and stored at –80°C for downstream assays.

### (c) Symbiont density

Symbiodiniaceae density was estimated using a standard haemocytometer and Nikon Eclipse E600 microscope. Symbiodiniaceae counts were repeated in triplicate and averaged to calculate symbiont density/tissue area.

### (d) Biochemical immune assays

Biochemical immune assays were conducted following established protocols for scleractinian corals, with minor modifications necessary to adapt the procedures for *A. poculata* [31–34]. Constitutive immunity was measured using assays estimating activity of the prophenoloxidase cascade (total phenoloxidase activity and melanin concentration), antioxidant activity (catalase and peroxidase) and antibacterial activity. All assays were standardized by either protein concentration or dry tissue weight, as appropriate. Assays were run in duplicates on 96 well plates using a Cytation 1 cell imaging multi-mode reader with Gen5 software (BioTek). Full assay details can be found in elecronic supplementary material, document 1.

### (e) Statistical analyses

Prior to statistical testing, outliers were identified and removed if necessary, using the ‘nooutlier’ function in R. Normality was assessed using a Shapiro test and homogeneity of variance was analysed using a levene test. The data were transformed as needed; Symbiodiniaceae density was square root transformed. We assessed the effects of symbiont density on each of our immunological metrics using two approaches. First, we tested for differences in assay activity between colonies grouped as white or brown using a *t*-test. Second, because symbiont density was highly variable within our groups, we also conducted correlative analyses (Pearson correlations) to look at direct correlations between symbiont density and activity assay. *t*-Tests and correlations were run independently for each assay. All raw data and code used are available on Dryad [35].

## 3. Results

**Figure 1.**
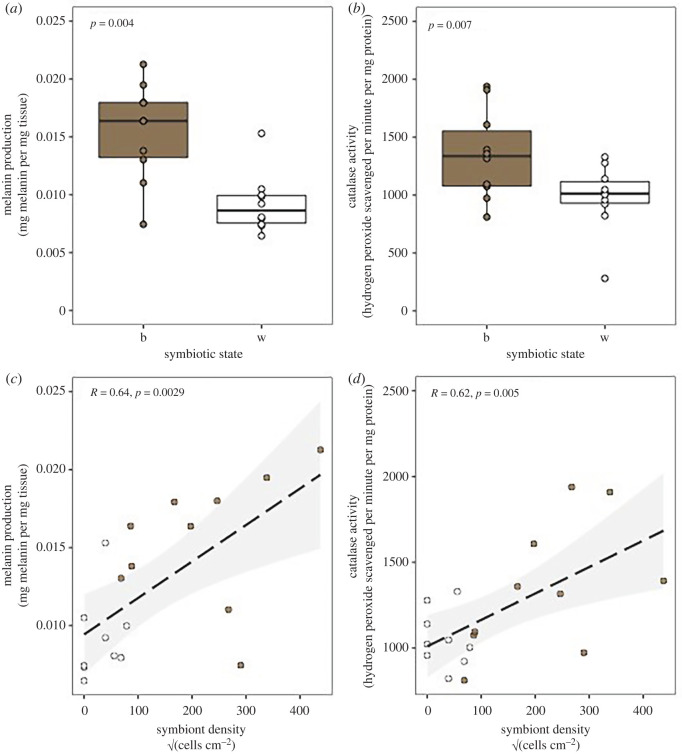
Both symbiont state (brown, white) and *Breviolum psygmophilum* density affect melanin concentration and catalase activity. (*a*,*b*) Box and whisker plots displaying differences in immune parameters between white and brown colonies for melanin (*a*) and catalase (*b*). (*c*) Melanin concentration and (*d*) catalase activity in symbiont immune assays, according to *B. psygmophilum* density.

Statistical analysis revealed a significant association between symbiotic state and host immune phenotypes. Both melanin concentration (*t*-test, *p* = 0.0004; figure 1*a*) and catalase activity (*t*-test, *p* = 0.048; figure 1*b*) were significantly higher in brown colonies than white. Furthermore, melanin concentration (Pearson correlation, *R* = 0.64, *p* = 0.003; figure 1*c*) and catalase activity (Pearson correlation, *R* = 0.62, *p* = 0.005; figure 1*d*) were significantly positively correlated to symbiont density. No other assays were significantly associated with symbiont state or symbiont density (tables 1 and 2).

**Table 1.** *t*-Test results for each immunological assay.

assay	statistic value	d.f.	*p*-value
peroxidase	−0.696	13.8	0.498
prophenoloxidase	−0.816	18	0.425
catalase	2.19	12.5	0.0482*
antibacterial	1.03	12.9	0.321
melanin	4.96	11.2	0.0004*

**Table 2.** Pearson correlation results between assay activity and square-root transformed symbiont density.

assay	corr. value	d.f.	*p*-value
peroxidase	0.2430729	17	0.316
prophenoloxidase	−0.0130134	18	0.9566
catalase	0.6155106	17	0.005026*
antibacterial	−0.06311574	18	0.7915
melanin	0.6900038	17	0. 0011*

## 4. Discussion

Here we used a facultatively symbiotic coral, *Astrangia poculata,* to investigate trade-offs between constitutive immunity and Symbiodiniaceae density in corals. Past studies have suggested trade-offs between the maintenance of symbiotic relationship and immunity in obligately symbiotic corals [27,29]. By contrast, our results show no trade-offs between Symbiodineaceae abundance and constitutive immunity. Instead, we find a positive association between constitutive immunity and Symbiodineaceae density in *A. poculata*. These findings confirm the complex nature of the relationship between algal symbiosis and immunity in cnidarians and highlight the need for further study of symbiosis–immune interplay in diverse systems.

Here we document positive correlations between symbiont density and two metrics of constitutive immunity: catalase activity and melanin concentration. Importantly, while both systems function in immunity, they also serve secondary roles in maintenance of coral–algal symbiosis [36]. While antioxidant activity is important in combating ROS bursts associated with pathogen defence, it is also important in general stress response, including response to thermal stressors [37]. Symbiont release of ROS is believed to be a cause of thermally induced bleaching, or breakdown of algal symbiosis [38]. Consistent with this theory, increased antioxidant production is associated with increased resistance to thermal bleaching [39]. Similarly, in addition to its roles in encapsulation of pathogens [12], melanin may play secondary roles in stress response, including protection of algal symbionts from UV damage (i.e. symbiont shading; [40]). Consequently, observed patterns of higher activity of these two pathways may be indicative of algal symbiont management and proactive stress mitigation mechanisms rather than direct consequences of symbiosis on immunity.

A second hypothesis could explain the observed associations between Symbiodiniaceae density and immunity more generally: resource allocation theory. Resource allocation theory posits that organisms allocate a fixed energetic budget to competing needs (ex: growth, reproduction and immunity; [41]). When energy budgets are fixed, increases in any one category come at the cost of another (i.e. trade-offs; [41]). Consequently, energetic budgets can have significant impacts on resources allocated to immunity. For example, reductions in energy budgets caused by starvation resulted in decreased expression of immune genes and resistance to pathogens in the cnidarian *Nematostella vectensis* [42]. Indeed, facultative symbiosis may be a natural source of variation in energetic budget; colonies of corals with variable densities of Symbiodiniaceae may vary in their base energetic budget due to increased photosynthetically derived carbon. Past studies have linked increased photosynthetic energy acquisition to increased Symbiodiniaceae density [43,44]. Consequently, increased *B. psygmophilum* densities in *A. poculata* may increase a colony's total energetic budget, allowing for greater resource allocation to immunity and explaining elevated catalase and melanin levels in colonies with higher *B. psygmophilum* density.

Regardless of mechanism, these findings add to a growing body of work considering the effects of symbiont density on immunity in cnidarians. Interestingly, previous work in obligately symbiotic corals suggests a negative relationship between symbiont density and immune gene expression [27,29], opposite to this study. A similar pattern was also observed in another obligately symbiotic coral, *Acropora tenuis;* immune gene expression was downregulated to allow for the establishment of symbiosis [45]. However, these previous studies involved obligately symbiotic corals, whereas our results describe patterns in a facultatively symbiotic coral. Variation in symbiont density, and therefore energetic budget, is likely more pronounced in the latter group, affecting our results. Additionally, the past studies applied broad transcriptomics approaches while this study only measured a handful of immune effector responses with dual roles in stress response and symbiosis maintenance. A broader approach might yield different results. Finally, in the Fuess *et al*. 2020 study, nutrient enrichment was used to artificially manipulate symbiont density [29]. Recent findings have suggested that nutrient enrichment may inhibit coral immune responses (PO activity) [46], suggesting this may have confounded results from the previous study. More studies investigating symbiosis–immune interactions in diverse cnidarian species using consistent approaches will be essential in disentangling this nuanced relationship.

In summary, our results highlight a positive association between *B. psygmophilum* density and immune parameters in the temperate coral *A. poculata*, which contrasts with past studies of obligately symbiotic corals. This association is most likely either related to the dual function of these parameters or a consequence of increased energetic budgets associated with symbiosis. Importantly, our approach only measured a subset of potential effector responses. Future studies incorporating more responses or measures of receptor and signalling activity would improve interpretation of these trends. Additionally, our results are limited to the context of constitutive immunity; further studies considering pathogen response would be informative. Finally, our results are limited to the context of common garden conditions; additional natural studies which highlight the immunological effects of interactions between symbiont density and environmental variation would be informative. Nevertheless, our data provides an important first step in highlighting the nuanced association between immunity and algal symbiosis in scleractinian corals.

## Data Availability

Raw data and code are available on Dryad Digital Repository: https://doi.org/10.5061/dryad.1ns1rn8xb [35].
